# Acute Myeloid Leukemia as a Model for Cancer Therapy

**DOI:** 10.3389/fonc.2012.00139

**Published:** 2012-10-12

**Authors:** Farhad Ravandi

**Affiliations:** ^1^Leukemia/Cancer Medicine, M. D. Anderson Cancer Center, University of TexasAustin, TX, USA

Up to 85% of adult patients with AML achieve complete response (CR) using current standard regimens and depending on pre-treatment characteristics. However, only ∼25–45% have a long-term leukemia-free survival depending their age and leukemia features. A number of clinical features such as age, performance status, organ dysfunction, and biological characteristics of the disease (most importantly cytogenetics) influence the outcome with several large studies showing the importance of karyotypic features of the leukemic blasts. Other prognostic indicators such as mutations or abnormal expression of specific genes such as *MLL*, *CEBPA*, *FLT3*, *NPM1*, *DNMT3A*, *TET2*, *ASLX1*, *IDH1*, *IDH2*, *WT1*, *MN1*, *BAALC*, and *ERG* have been recently described and are particularly useful in assigning risk to patients with diploid AML (intermediate risk cytogenetics group). Clearly, these data support the notion that neoplastic change is the culmination of a number of molecular events that result in deregulated cellular pathways with more molecular aberrations leading to diseases that are more resistant to traditional cancer therapeutics. Therefore, new treatment strategies are needed particularly true for patients with non-favorable risk features.

Despite major advances in understanding the biology of specific subtypes of leukemia, the standard therapy for patients with AML has changed little over time. A notable exception has been in the treatment of patients with acute promyelocytic leukemia (APL) with the introduction of all-trans retinoic acid (ATRA) and arsenic trioxide (ATO), drugs targeted at the specific molecular aberration causative of the disease [the PML-RARA fusion product of *t*(15;17) translocation, pathognomonic for the disease]. Several studies using “chemotherapy-free” regimens combining ATRA, ATO with or without gemtuzumab ozogamicin (GO) have produced high and durable response rate. Although the combination of cytarabine and an anthracycline continues to be the basis for most induction and consolidation regimens in other types of AML, development of specific drugs targeting molecular aberrations such as mutated FLT3, RAS, and the PI3K signaling pathways are showing the way for potential personalized therapy in the future.

Better understanding of the biology of AML has identified new targets for therapeutic intervention. One of the best examples of this is the identification of the mutated form of FLT3 tyrosine kinase which confers a worse outcome in the 30% of patients whose leukemic cells bear this abnormality. Several inhibitors of FLT3 kinase have been developed and evaluated at our institution including lestaurtinib, sorafenib, and quizartinib with the latter two drugs demonstrating significant activity. Other potential targets include *NPM1* (reports suggesting a role for ATRA in *NPM1* mutated diploid AML), c-*KIT* in CBF leukemias, the JAK-STAT signaling pathway in post-MPN AML, the MAPK pathway in *RAS* mutated leukemias and various cellular signaling components which include a number of tyrosine and serine/threonine kinases. This ever expanding list of potential targets has advantages and disadvantages in terms of therapeutic intervention. The advantages include well defined hypotheses that can be tested using correlative studies to determine whether the inhibition of the specific target or pathway can be beneficial clinically. The potential problem comes from the fact that many of these targets are redundant and overlapping and as such their specific inhibition may not lead to meaningful benefits despite their significant role in leukemic cell survival. It is also clear from early studies that the many of these agents are able to achieve more meaningful responses when combined with traditional agents, such as cytarabine and anthracyclines or novel agents such as the hypomethylating compounds. They are also likely to have a major role in the post-remission/consolidation setting where they can and should be evaluated for the eradication of minimal residual disease (MRD).

Better evaluation of MRD and a better understanding of the biology of the leukemogenic cells (“Leukemia stem cells”) may allow the development of new agents with alternative mode of action active against these residual cells. Relapse in AML is the result of the resurgence of leukemic cells that have escaped induction and consolidation therapy and have persisted in complete remission (CR). This MRD is not detectable by the standard techniques used to define CR; therefore, more sensitive techniques such as polymerase chain reaction (PCR) and multi-parameter flow cytometry (MFC) are used increasingly to define MRD. PCR-based strategies to detect MRD have been largely limited to patients with favorable risk disease where the fusion transcripts *AML1-ETO*, *CBFB-MYH11*, and *PML-RARA* can be monitored. This proportion can be potentially increased using the newly identified mutated genes such as *FLT3 and NPM1* as well as over-expressed genes such as *WT1*. MRD monitoring with flow cytometry relies on the idea that AML cells frequently have aberrant antigen expression resulting from asynchronous antigen-expression, cross-lineage antigen expression, antigen over-expression, and aberrant light scatter properties. Early studies of immunophenotypic detection of MRD in AML were based on double antigen staining analyzed by fluorescence microscopy. More recent studies have demonstrated that using MFC, monitoring may be applicable to virtually all patients and allow a more precise detection of the MRD with aberrant phenotype. Such immunophenotypic evaluation of MRD by MFC has been demonstrated to be useful in predicting the risk of relapse after achieving CR. The identification of leukemia stem cells in this setting, using specific markers, would be particularly attractive as it may allow the application of specific agents with the ability to target and eradicate these leukemia-sustaining cells.

We have seen limited progress in the treatment of the complex disease, AML, over the past several decades. Recent progress suggests that AML therapy can lead the way to unraveling the complexities of cancer therapy as a whole and lay further foundations to enhance our understanding of carcinogenesis and cancer therapy. Similar progress in the understanding of the molecular pathogenesis of other hematological neoplasms as well as solid tumors is opening the doors toward the development of more effective and less toxic target specific agents and will no doubt further change the strategies of combating and curing these disorders. Small molecule inhibitors of components of various deregulated signaling pathways as well as monoclonal antibodies and immune based therapies have already dramatically altered treatment algorithms in lymphomas, leukemias, and myeloma with further highly effective agents continuing to be developed.

In this journal we are hoping to capture the interest of the readers interested in keeping up with these advances through publishing up-to-date, and concise review articles summarizing the advances in the field as well as original articles which are helping to unravel the mysteries of carcinogenesis and leading to the new therapeutic advances.

